# Markers of cerebral damage during delirium in elderly patients with hip fracture

**DOI:** 10.1186/1471-2377-9-21

**Published:** 2009-05-27

**Authors:** Barbara C van Munster, Catharina M Korse, Sophia E de Rooij, Johannes M Bonfrer, Aeilko H Zwinderman, Johanna C Korevaar

**Affiliations:** 1 Department of Clinical Epidemiology. Biostatistics and Bioinformatics, Academic Medical Centre, University of Amsterdam, P.O. Box 22660, 1100 DD Amsterdam, the Netherlands; 2 Department of Medicine, Academic Medical Centre, University of Amsterdam, P.O. Box 22660, 1100 DD Amsterdam, the Netherlands; 3 Department of Clinical Chemistry, Netherlands Cancer Institute, P.O. Box 90203, 1006 BE Amsterdam, the Netherlands

## Abstract

**Background:**

S100B protein and Neuron Specific Enolase (NSE) can increase due to brain cell damage and/or increased permeability of the blood-brain-barrier. Elevation of these proteins has been shown after various neurological diseases with cognitive dysfunction. Delirium is characterized by temporal cognitive deficits and is an important risk factor for dementia. The aim of this study was to compare the level of S100B and NSE of patients before, during and after delirium with patients without delirium and investigate the possible associations with different subtypes of delirium.

**Methods:**

The study population were patients aged 65 years or more acutely admitted after hip fracture. Delirium was diagnosed by the Confusion Assessment Method and the subtype by Delirium Symptom interview. In maximal four serum samples per patient S100B and NSE levels were determined by electrochemiluminescence immunoassay.

**Results:**

Of 120 included patients with mean age 83.9 years, 62 experienced delirium. Delirious patients had more frequently pre-existing cognitive impairment (67% vs. 18%, p < 0.001). Comparing the first samples during delirium to samples of non-delirious patients, a difference was observed in S100B (median 0.16 versus 0.10 μg/L, p = < 0.001), but not in NSE (median 11.7 versus 11.7 ng/L, p = 0.97). Delirious state (before, during, after) (p < 0.001), day of blood withdrawal (p < 0.001), pre- or postoperative status (p = 0.001) and type of fracture (p = 0.036) were all associated with S100B level. The highest S100B levels were found 'during' delirium. S100B levels 'before' and 'after' delirium were still higher than those from 'non-delirious' patients. No significant difference in S100B (p = 0.43) or NSE levels (p = 0.41) was seen between the hyperactive, hypoactive and mixed subtype of delirium.

**Conclusion:**

Delirium was associated with increased level of S100B which could indicate cerebral damage either due to delirium or leading to delirium. The possible association between higher levels of S100B during delirium and the higher risk of developing dementia after delirium is an interesting field for future research. More studies are needed to elucidate the role of S100B proteins in the pathophysiological pathway leading to delirium and to investigate its possibility as biomarker for delirium.

## Background

Postoperative delirium following orthopedic surgery for hip fracture occurs in up to 62% of the elderly patients, many with pre-existing dementia[1]. It is characterized by fluctuating changes in cognition, consciousness and attention and lasts on average, three days[1,2]. Three clinical subtypes of delirium are known; a hyperactive, a hypoactive, and a mixed subtype [3,4]. So far, the suggested pathophysiological mechanisms of delirium are mostly hypothetical, but lately there is a lot of interest in the neuroinflammatory system [5-7]. Although patients usually recover after resolution of the underlying cause, delirium appears to be an important risk factor for dementia, even in people without prior cognitive impairment[8].

The high frequency of dementia after delirium possibly reflects irreversible brain damage caused by the detrimental effects of the pathophysiological mechanisms of delirium on the brain. Moreover, it can be hypothesized that postoperative delirium itself might be the consequence of transient cerebral neuronal damage caused by peri-operative hypoxia, micro embolisms[9] or hypotension[10].

S100B-protein (S100B) and Neuron Specific Enolase (NSE) have been used as markers of brain damage in various diseases[11]. S100B belongs to the family of calcium binding proteins, it is expressed mainly by astrocytes and it is found in both intra- and extracellularly in brain tissue[12]. S100B is usually elevated in blood and in CSF due to nervous system damage, via functional disturbance of membrane integrity and/or increased permeability of the blood-brain barrier. NSE is the intracytoplasmic glycolytic enzyme enolase. It is found in neurons and neuro-endocrine tissue and it is elevated in the blood circulation after increased death rate of these cells.

Elevated levels of S100B have been shown to be associated with delirium in patients after abdominal surgery[13], after cardiac surgery[14] and in sepsis-associated delirium[15]. NSE was not elevated in serum in patients with delirium tremens[16] but it has been found to correlate with the degree of cognitive dysfunction at discharge from hospital after cardiac surgery but not after non-cardiac surgery[17,18]. In these previous studies the delirious state at the day the sample was taken was not taken into account, neither subtype. The aim of this study in elderly hip fracture patients was twofold: (1) to compare changes before and after surgery of S100B and NSE levels in serum in patients with and without postoperative delirium, and to investigate the difference in serum levels before, during and after delirium; (2) to study the serum levels of S100B and NSE in different subtypes of delirium.

## Methods

### Patients

From May 2005 through February 2008, all consecutive patients 65 years of age or older who were acutely admitted with hip fracture to the Academic Medical Centre, Amsterdam were invited to participate. Informed consent was obtained from all patients or from substitute decision-makers in cases of cognitive impairment. Patients were excluded if they were not scheduled for operation or were unable to speak or understand Dutch or English. The institutional Medical Ethics Committee approved the study.

### Procedures

Members of the research team completed a multidisciplinary evaluation for all study participants. Two geriatricians, a fellow in geriatric medicine, and four research nurses trained in geriatric medicine collected demographic and clinical data. The presence or absence of delirium was scored during weekdays separately by a physician and a nurse using the Confusion Assessment Method (CAM) [19]. We based our information for the diagnosis on our psychiatric examination of the patient, medical and nursing records including the Delirium Observation Screening Scale (DOS) [20], and information given by the patient's closest relative. When the diagnosis of delirium was doubtful, the patient was discussed in the geriatric consultation team to gain consensus. To identify the subtype of delirium we used the Delirium Symptom Interview with the cut-off scores described by Liptzin et al This resulted in three different subtypes: the hyperactive, hypoactive and mixed subtype[21]. The Delirium Rating Scale-Revised-98 (DRS-R-98) was used to monitor the severity of delirium[22,23].

Possible confounding factors, including demography, fracture characteristics, type of anesthesia, type of surgery, and peri- and postoperative complications were registered for all patients. The number of medication taken at home was taken as global measure of co-morbidity. Pre-existent global cognitive functioning was based upon anamnesis, medical history, and IQCODE-SF (Informant Questionnaire on COgnitive Decline-Short Form). The IQCODE-SF assesses the possible presence of global cognitive impairment based on the response of an informant who had known the patient for at least ten years [24]. The informant was asked to recall the situation two weeks prior to the hip fracture and compare it with the situation ten years earlier. Patients with a mean score of 3.9 or higher were considered to have global cognitive impairment [25]. To measure pre-existent physical functionality, we asked patients, or their closest relative in cases of cognitive impairment, to complete the 15-item Katz ADL scale based on the situation two weeks prior to the hip fracture[26].

A maximum of four blood samples was collected during the hospital stay under similar conditions around 11.00 am. Samples were taken during weekdays whenever patients were available for venipuncture. Blood was kept on ice after withdrawal. Serum was obtained by centrifugation for 15 minutes at 1780 g at 4°C, and aliquots were stored at -80°C. S-100B and NSE levels were measured on the Modular Analytics E170 (Elecsys module) analyzer (Roche Diagnostics, Mannheim) using the electrochemiluminescence immunoassays (ECLIA) technique. Normal values were established for S100B as below 0.10 μg/l and for NSE as below 12.5[27,28]. NSE will not be determined in case of even light hemolysis, because hemolysis could substantially increase the NSE value in serum due to the presence in erythrocytes[12]. Interleukin (IL) concentrations (IL-6 and IL-8) were measured using a cytometric bead array (CBA) immunoassay (BD Biosciences Pharmingen, San Diego, CA). The chemical analysts were blinded to the clinical diagnosis of the patients.

### Statistical Analysis

Statistical Package for the Social Sciences (SPSS) version 15.0 was used for data analysis. We tested for differences in characteristics in patients with and without delirium using T-tests and Mann-Whitney Tests. Variables that were not normally distributed were expressed as median scores and inter-quartile ranges. Differences in NSE levels in different subtypes were analyzed using ANOVA and in S100B levels using the Kruskal-Wallis test. A two-tailed criterion of p < 0.05 was considered statistically significant. Correlations between NSE and DRS-R-98 score, between S100B and DRS-R-98 score, and between S100B and NSE with IL-6 as well as IL-8 were determined using Spearman's rho test.

To examine the association of S100B with delirium state we used the linear mixed models approach. For S100B level the e-based log transformation was used, patient number was taken as a random effect, and day of sampling, delirious state, and the interaction between these two variables were taken as fixed effects. The association between NSE and delirium was analyzed in a similar way 'Delirious state' consisted of four categories based on the CAM scores of the patient at the moment the sample was taken: samples from patients in the non-delirious group were all categorized (1)'non-delirious', samples from patients in the delirious group were categorized (2)'before delirium', (3)'during delirium' or (4)'after delirium'. In addition, pre- or postoperative status, type of fracture, type of surgery, type of anesthesia, global cognitive impairment, and age were considered as covariates in the mixed model. In a backward selection procedure within the linear mixed models all variables with p-value above 0.05 were removed. The goodness of fit of the mixed models was inspected by assessing the distribution of the residuals.

## Results

During the inclusion period 271 patients aged 65 years and older were admitted. A total of 55 patients were excluded due to lack of informed consent, 63 patients did not consent to extra blood withdrawal and 33 patients were excluded for logistic reasons. In and excluded patients were similar with regard to the male/female ratio, but the excluded patients were younger (mean age of 81.3 years versus 83.9 years, p = 0.005). In total 120 patients were included, 62 (52%) with delirium and 58 without. This resulted in 387 samples in total, 196 samples of delirious patients (21 samples taken before the delirious episode, 120 samples taken during the delirious episode, 55 taken after the delirious episode) and 191 samples of patients without delirium.

Patients with delirium experienced significantly more often pre-existent cognitive and functional impairment (p < 0.001) and lived in either old-people's homes or nursing homes significantly more frequently than non-delirious controls (p < 0.001) (Table 1). There was no significant difference in the type of anesthesia (p = 0.35), type of surgery (p = 0.54) or total number of complications (p = 0.19) between patients with and without delirium. The median time of admission was 14 days for patients with delirium and 11 days for patients without delirium (p = 0.04).

**Table 1:** Characteristics of Patients With and Without Delirium.

Variables	Delirium (N = 62)	No delirium (N = 58)	p-value
Age (yrs)	84.8 (6.9)	82.9 (7.0)	0.14
Male gender (%)	16 (26)	23 (40)	0.11
Living at home (%)	30 (48)	51 (88)	< 0.001
Pre-admission functional impairment (number of ADL disabilities)	8 (6–11)	4 (2–6)	< 0.001
Number of medication at home	5 (2–7)	4 (2–6)	0.18
Pre-admission cognitive impairment (%)	39 (67)	10 (18)	< 0.001
Days between fracture and operation	1 (0–1)	1 (1–2)	0.49
Spinal anesthesia (%)	22 (36)	19 (33)	0.35
Fracture characteristics (%)			0.54
Femoral neck	25 (40)	28 (48)	
Intertrochanteric	33 (53)	25 (43)	
Other	4 (7)	5 (9)	
Type of surgery (%)			0.64
Internal fixation	40 (65)	35 (60)	
Hip replacement	22 (36)	23 (40)	
Complication (%)-total	29 (50)	22 (38)	0.19
IL-6 (pg/ml)*	51 (28–88)	36 (14–58)	0.01
IL-8 (pg/ml)*	15 (7.9–37)	9.3 (4.6–17)	0.03
Admission length	14 (8–23)	11 (8–14)	0.04
Death within 3 month (%)	11 (18)	5 (9)	0.14

Mean values (SD) are given for continuous variables with a normal distribution,

Median values (inter-quartile ranges) are given for variables that are not normally distributed.

ADL: Activities of daily living;

*Values of 47 delirious patients during delirium vs. 48 patients without delirium.

Table 2 describes the levels of S100B and NSE in the first sample during the delirious episode in the delirious group compared to samples withdrawn at a comparable day before or after surgery of the non-delirious group (mean postoperative day 1.3, SD: 1.6). More delirious (81%) than non-delirious (50%) patients had levels above the normal values of S100B (p = 0.001). Also, the levels of S100B were higher in delirious patients (0.16 μg/L) compared to the non-delirious patients (0.10 μg/L) (p < 0.001). In our samples NSE was not determined in 153 (38%) of the samples, because of hemolysis due the preservation of the samples on ice after withdrawal. The NSE level was 11.7 (ng/L) both in cases and controls (p = 0.97). There was no significant correlation with S100B and NSE levels length of admission, death within the first 3 month or preoperative cognitive impairment. No correlation between NSE levels and IL-6 or IL-8 was observed, but significant correlation was found between levels of S100B and Il-6 (r = 0.35, p < 0.001) or IL-8 (r = 0.36, p < 0.001).

**Table 2:** S100B (μg/L) and NSE (ng/mL) values during delirium versus no delirium.

	During delirium (N = 57*)	No delirium (N = 58)	p-value
NSE – number	35	40	
N (%) > 12.5	9 (26)	15 (38)	0.28
Mean (SD)	11.7 (3.8)	11.7 (4.7)	0.97
S100B – number	57	58	
N (%) > 0.10	46 (81)	29 (50)	0.001
Median (IQR)	0.16 (0.10–0.25)	0.10 (0.08–0.15)	< 0.001

*In the group with delirium samples were the first samples during the delirious episode; in the group without delirium, samples of comparable postoperative day were taken. Of five patients in the delirious group no sample taken during delirium.

**Figure 1:**
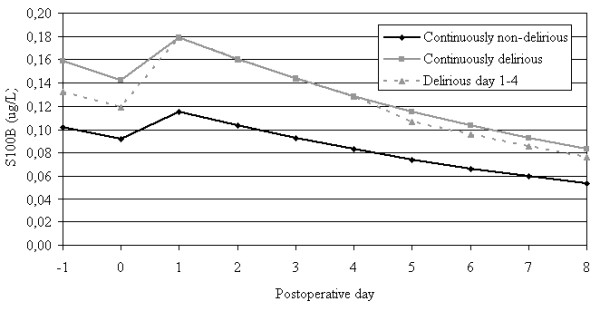
**Three representative patients with an intertrochanteric fracture to show the changes of S100B during admission calculated with values of the mixed model analysis of all samples**. The final model included delirious state (p < 0.001), day of blood withdrawal (p < 0.001), pre- or postoperative status (p = 0.001) and type of fracture (p = 0.036). Postoperative day 0 is day of surgery.

To study the course over time of S100B, we included samples from the day before surgery until post-operative day eight because during this period samples from both delirious and non-delirious patients were available. Following a backward approach, type of surgery, type of anesthesia, global cognitive impairment, and age were removed. The final statistical model included delirious state (p < 0.001), day of blood withdrawal (p < 0.001), pre- or postoperative status (p = 0.001) and type of fracture (p = 0.036). To illustrate the effect of delirium on S100B, levels of three different example patients with an intertrochanteric hip fracture were calculated and are presented in figure 1: 1) a patient who was delirious from one day before operation until eight days after operation (continuously delirious), 2) a patient who was never delirious during this period (continuously non-delirious), and 3) a patient who was delirious from the day after operation until postoperative day four (delirious day 1–4). S100B levels were highest 'during' delirium at all time-points. 'Before' and 'after' delirium S100B levels were still higher than those from 'non-delirious' patients. Delirious state was not significant in the same mixed model analysis with NSE in 233 samples. Spearman's rho test showed no significant correlation between S100B and NSE levels in the first sample (p = 0.99).

In 34 delirious patients, delirium subtype was specified; one patient did not meet the criteria for any of the subtypes (Table 3). Specification of the subtype was missing for 23 delirious patients and from five patients no blood sample was taken during delirium. Delirious patients with known subtype were similar to delirious patients with unknown subtype with respect to age, sex and cognitive impairment, day of blood withdrawal, pre- or postoperative status and type of fracture. No large difference was observed in NSE and S100B levels in patients with different subtypes, nor was there a difference in levels between patients with known vs. unknown subtype. Of 38 patients the severity of delirium (DRS-R-98) was specified. Spearman's test revealed no significant correlation between levels of DRS-R-98 and NSE (r = 0.20, p = 0.32) or S100B (r = -0.08, p = 0.62).

**Table 3:** Values of NSE (ng/mL) and S100B (μg/L) and DRS-R-98 scores during delirium in different subtypes.*

	Hyperactive	Mixed	Hypoactive	p-value
NSE – number	11	8	4	
N (%) > 12.5	4 (36)	4 (50)	1 (25)	0.68
Mean (SD)	12.2 (3.8)	14.3 (5.5)	11.0 (2.8)	0.41
				
S100B – number	17	12	4	
N (%) > 0.10	13 (77)	11 (92)	3 (75)	0.54
Median (IQR)	0.17 (0.10–0.21)	0.23 (0.11–0.32)	0.12 (0.09–0.54)	0.43
				
DRS-R-98†	19	20	18	0.25

IQR: inter-quartile-range; DRS-R-98: Delirium Rating Scale-R-98.

*Subtype is specified in 34 delirious patients; one patient did not meet the criteria of any of the subtypes; specification of the subtype was missing for 23 delirious patients; of five patients no blood sample was taken during delirium.

†DRS-R-98 score is specified in 29 of the patients with known subtype.

## Discussion

This study among elderly patients admitted for hip fracture showed that delirium was associated with increased level of S100B which could indicate cerebral damage either due to delirium or leading to delirium. The study population represents a typical cross-section of elderly patients admitted for hip fractures. A similar frequency of delirium (52%) has been found in other large studies of hip fracture patients[29]. The expected risk factors for delirium in surgical patients, such as functional and cognitive impairment, were confirmed in our study[30]. Next to delirium, type of fracture, pre- or postoperative status and day of blood withdrawal were all associated with S100B levels. The effect of fracture and surgery and the time course were expected covariables based on the literature[31]. Although the group of delirious patients with known subtype was small, the hyperactive type of delirium was the most common subtype of delirium in our study, similar to what has been described by others[32]. Moreover, selection bias seems improbable since the delirious patients included in this analysis did not differ in associated factors with S100B and levels of NSE and S100B compared to the delirious patients without known subtype. Because this still could have introduced a bias, future studies are needed to confirm the lack of association between subtypes and NSE or S100B.

A limitation of our study is that the NSE and S100B values were measured from peripheral blood and may not necessarily correspond to values in the brain. Under normal conditions serum NSE and NSE in cerebrospinal fluid (CSF) show no significant correlation, whereas serum S100B content is lower than that in CSF[11,33]. We are not aware of studies that determined NSE and S100B in both CSF and blood among patients with cognitive deficits such as delirium. A second limitation of our study is that only 120 of the 183 (66%) patients consented to multiple blood withdrawals. Although the excluded patients were 2.5 yrs. younger it is unlikely that this small difference would affect the protein levels.

Our results showing elevated S100B levels in delirious patients are in line with former studies [13-15]. Studies looking at postoperative cognitive deficit (POCD) and S100B after surgery show however contradicting results, with no relation between S100B and cognitive performance after cardiac surgery at discharge [17], a questionable relation directly after cardiac surgery [34], but higher levels of S100B in patients with POCD after non-cardiac surgery[18]. The definition of POCD remains controversial, but it seems likely that delirium and short-term POCD are on a continuum of postoperative cognitive brain disorders[35]. Seeing delirium as subset of POCD, our results of NSE correspond to former studies that found no association between NSE and POCD at discharge from hospital after non-cardiac surgery[18]. Maybe NSE is released only in cases of severe brain damage, such as stroke, after resuscitation or neurologically complicated surgery[18].

Importantly, S100B is a marker of cerebral damage and/or reduced integrity of the blood brain barrier. In stroke, global hypoxia and traumatic brain injury a positive correlation between S100B and cognitive outcome has been established [36]. Possibly, the high frequency of dementia after delirium could also be predicted by the level of S100B during delirium. Predictive factors for cognitive outcome after delirium are unknown, largely because of the difficulties of diagnosing pre-existent cognitive functioning in patients with delirium[5]. On the one hand delirium and dementia (or cognitive impairment) could each be caused by the same precipitating factors such as the surgical procedure[7]. On the other hand, it could be hypothesized that dementia is caused by the detrimental effects of delirium itself [5]. The neuroinflammatory system seems to be a rational pathophysiological pathway for delirium [5-7] and this same system has been described in relation to cerebral damage [11]. The correlation between the levels of S100B and the proinflammatory cytokines IL6 and IL8 in the patients in this study can be seen as an indication that the neuroinflammatory system is related to possible cerebral damage due to delirium.

## Conclusion

In our study of elderly patients admitted for acute surgical repair of hip fracture we found higher S100B levels in patients with delirium than in patients without delirium with the highest levels of S100B during delirium,. No difference in S100B and NSE levels between the different subtypes of delirium was found. Future studies are needed to elucidate the place of S100B in the pathophysiological pathway leading to delirium (and possibly dementia) and investigate its role as biomarker for delirium.

## Competing interests

The authors declare that they have no competing interests.

## Authors' contributions

BM and SR performed the data collection. CK and JB carried out the immunoassay. BM performed the statistical analysis under supervision of JK and AZ. All authors read and approved the final manuscript.

## Pre-publication history

The pre-publication history for this paper can be accessed here:


